# Dr. Patterson, on Chorea Sancti Viti

**Published:** 1806-02

**Authors:** Wm. Patterson

**Affiliations:** Londonderry


### To Dr. BRADLEY.

Dear Sir,

In the 13th volume of the Medical and Physical Journal, page 401, is inserted a paper, entitled "Observations on Chorea Sancti Viti, with a new Theory of the Disease; by John Redman Coxe, M. D. of Philadelphia." The *new theory*, propounded in that paper, is, that chorea is *merely* a symptomatic affection of a disease, namely, hydrocephalus internus, which, though long known, has not, in Dr. Coxe's opinion, been thoroughly understood till of late years. Whether Dr. Coxe's theory be new or old, whether it be true or false, I should not have taken the time thus professedly to examine, had he not referred to a publication tion of mine on the internal dropsy of the brain, and censured not only some of my reasoning, but also part of my practice, in a manner at once abrupt, rough, and magisterial. "It appears to me," says Dr. Coxe, "that Dr. Patterson has greatly erred in supposing chorea the primary disease; of which each must judge for himself." And adverting to the treatment of the interesting case, that of a young lady, for which he is indebted to my treatise, he further remarks, "The operation of the emetics probably hastened her dissolution, by determining too forcibly the circulation of the brain, (as Dr. Coxe expresses it), and hence exciting an acute state of hydrocephalus."

Before Dr. Coxe pronounced, that I "greatly erred in supposing chorea the primary disease," surely it behoved him to refute the reasoning whereon my conclusion is founded; yet he has not made the smallest atttempt at an undertaking, the performance of which is so necessary to vindicate his dogmatism. I shall, therefore, in justice to myself, and for the sake of truth and science, here transcribe my reasoning, and call upon Dr. Coxe for refutal, or for concession.

"With respect to the manner in which convulsive affections occasion a watery effusion in the brain, it may be observed, that persons liable to those affections exhibit a considerable degree of excitability, and that the principle of irritability is quickly accumulated and quickly diminished in their constitutions. Hence are derived the periodical or repeated paroxysms of those disorders, which the innate nervous stimulus on the one hand, and the prompt renovation of the irritable principle on the other, will naturally constitute.

"This habit is remarkably prevalent in childhood and youth; periods which compose the spring-time of life. And as in the vernal season, the vegetation of plants, which also depends on the mutual effects of stimulus and irritability, is in the greatest vigour; so in young persons, the same principles have their highest power, and are the agents of growth which conduct the species to its state of maturity. The principle of irritability is not confined to the fibre, or simple solid; but is also possessed by the fluids of the body, particularly by the blood. The heart, which contains so much of the fibre, and is the fountain of the fluid, must be the centre of the principle, and the influence of this organ on the contents of the cranium is well known.

"As in those ages, in which this constitution prevails, convulsions convulsions likewise prevail, the increased action which they produce will augment the impetus of the circulation, especially in the vessels of the head; which we see is actually the case, since a considerable determination to that part is observed in those disorders. This determination increases the action of the blood-vessels, and inflammation is the consequence. This consequence is forwarded by the plethora, particularly of the head, which exists in the early and advancing stages of life; and the whole train of symptoms, constituting *febricula hydrocephalica*, (the name which I appropriate to the disease), is then brought into view.

"But how are we to explain the occurrence of effusion, where the accumulation of the irritable principle is not so vigorous, and yet the action of stimuli is assiduous? In these cases I would conjecture, that the nervous stimulus, acting, as I suppose, on a mass of irritability less moveable than in convulsive habits, excites a passion, which, if violent, suddenly destroys irritability, or the vital principle, and death ensues; or which, if moderate, engages only a part of the irritability, and the natural state of temper is regained for a time. To illustrate this reasoning by an example: In an account of the death of the celebrated Mirabeau, we are told, 'Un temperament ardent et trop exercè a abregé les jours de cet homme célébre dont les talens faisoient l'esperance des amis et de la constitution.' Upon the report of poison having been administered, the body being opened, 'On a trouvé un épanchement dans un des lobes du poumon, et un amas de pus dans le pericarde de cœur; il y avoit aussi un petit épanchement dans *le cerveau*, tous les autres viscères étoient en bon état.' Does not this instance shew, that an irritable temper, almost constantly exercised, may be a cause of effusion in different cavities, and of a watery effusion in the brain; which conditions, in all probability, were preceded by more or less inflammation in the diaphanous membranes of those parts? And must we not admit an irritability of temper in those persons peculiarly subject to hydrocephalus internus?

"But, howevsr seasonable we may suppose the theory, we should not suffer it to engross our thoughts so much as to divert us from attending to any opportunity that may appear likely to advance the practice; especially in a disease delusive in its nature, alarming in its course, and too, too often fatal in its termination."* With

* Letters concerning the internal dropsy of the brain, p. 82—8 Edit. 1794.

With regard to the emetics, whose operation, Dr. Coxe says, probably hastened the death of the patient, a sense of candour, had it been felt, would have induced him to absolve the medical attendant from any blame on that score; for he might have observed, that those remedies were had recourse to by domestic advice, in the absence of the physician, who was at too great a distance to prescribe with that expedition which the existing circumstances were supposed to require. But even had the physician ordered, or sanctioned, those emetics, conceiving the case to be hydrocephalic, he would have had the authority of eminent professional men to support the practice. If the stomach appeared loaded, the experienced Dr. Fothergill gave a quarter or half a grain of tartar emetic, and afterwards appeased the vomiting with saline, absorbent medicines, occasionally adding a few drops of thebaic tincture. Dr. Warren, of Taunton, thinks that the *frequent use* of gentle emetics would probably afford relief; as emetics, he remarks, are known to be highly salutary in promoting absorption from cavities. In his opinion, they would certainly be safer than mercury, their operative action being soon over. Dr. Quin, of Dublin, says, "the *first step* to be taken is to empty the stomach," for which he assigns his reasons, but advises the mildest emetics, such as infusion of camomile, and cautions against the employment of antimonials. Dr. Aery, of Whitehaven, and Dr. Campbell, of Hereford, began the treatment with emetics; and Mr. Jameson, of London, gave an emetic, in the case of a girl, nine years old, the second day after serious symptoms of hydrocephalus internus appeared. To these may be added, Mr. Shaw, of Dudley in Worcester, Mr. Davis, of the Tower, &c. &c.*

In this case of my patient, therefore, had it been a case of hydrocephalus internus, and had I prescribed emetics, either in the early or advanced stage of the disease, I should have had sufficient precedents for the measure. But, in the irritable and reduced state of her stomach, I considered those Herculean emptiers, emetics, unwarrantable expedients; and had I viewed the disease in the light of idiopathic hydrocephalus internus, Dr. Coxe might have perceived, (page 35 of my tract) that my sentiments are

* London Med. Observ. and Inquir. Vol. IV. Art. III Simmons's Med. Journal, Vol. IX. Quin's Treatise on the Dropsy of the Brain, p. 63, 64. Edinburgh Med. Comment. Vol. VIII. p. 333, Id. Vol. IX. p. 240. London Med. and Physic. Journal, Vol. III. p. 517. Id. vol. VIII. p. 98.

are against emetics in diseases of a febrile cast, having a peculiar determination of blood to the head, such as genuine internal dropsy of the brain. No person, however, without distorting facts and perverting reason, could construe the case of my patient into a case of acute hydrocephalus: for, until the last short period of the illness, when the metamorphosis was brought round, the phenomena were of a very different character from those belonging to the hydrocephalus internus; and the watery effusion, which finally occurred in the ventricles of the brain, was similar to that which happens at the close of other distempers; for instance, in typhoid fever, in mania, and, what is more to our point, in epilepsy, a species of termination which shall be particularly noticed in the sequel.

The influence of convulsive affections in causing accumulations of the fluids in the brain is not a new point in pathology; it has occupied the attention of physicians during some years past. A child was indisposed about two months with frequent head-ach, which was supposed to proceed from worms, but anthelmintic medicines afforded no relief, and he died in a convulsive fit. On opening the head, the vessels of the brain were observed to be uncommonly turgid, and in the ventricles was found more than double the ordinary quantity of serum. "In this case," says the late Dr. Percival, of Manchester, "I apprehend the turgescence of the vessels was the *effect*, and *not the cause*, of the convulsions; for the reflux of the blood from the head to the heart being obstructed during the fit, in which I believe the patient expired, the vascular distension must have been permanent. The redness and even the blackness of the face, which takes place in convulsions, affords sufficient proof of sanguinous accumulations."* Hence it appears, that the same speculative opinion, which Dr. Coxe styles a *new theory*, with respect to convulsive motions in hydrocephalus, was conceived at least thirteen years before Dr. Coxe wrote, and had obtained so much credit as to induce the learned Dr. Percival to counteract its extension.

If, as Dr. Coxe supposes, chorea be not an idiopathic disease, but arises *solely* as a symptom of the *chronic* hydrocephalus," the characteristic phenomena of the latter should always precede, or accompany, the appearance of the former. Whereas, upon a careful examination, we shall

* Medical Tracts, Vol. 1. page 125, published in 1791.

shall find, that the symptoms of chorea exist without any token of hydrocephalus, as strikingly exemplified in the case of my patient, already alluded to, in whom its essential features prevailed long before it wrought the change which terminated in the fatal effusion into the ventricles of the brain. Had the cases of chorea, which came under the cognizance of Sydenham, been accompanied with hydrocephalic symptoms, so careful an observer certainly would have mentioned such striking occurrences, and would not have found, as he did, those cases liable to periodical returns, which rarely, or never, happen in any species of hydrocephalus.* Dr. Coxe, indeed, seems to be aware of the difficulty that would arise to him in this point, and puts the question, "If hydrocephalus is the cause of chorea, it may be asked, as the former is not an unfrequent disease, why the latter does not more often occur?" He confesses, that he is unable to resolve the question, because he includes himself among those who know too little of the nervous system to reason accurately upon it; but tries the point with another query, "Why the apoplexy, epilepsy, palsy, &c. are not always produced?" To this I answer, surely they always would be produced, if they were symptoms of hydrocephalus; and so would chorea, if it possessed the affinity of a symptom.

In the second case of chorea, stated in my Tract, p. 77, not a symptom of hydrocephalus can be traced; of the same description is Dr. Coxe's second case; and many more cases are on record, wherein the former disease has been totally independant of the latter. Instead of being solely a symptom of chronic hydrocephalus, chorea sometimes proceeds from obstructed menses, sometimes from exposure to rigorous weather, but generally from irritation in the first passages. So strong a tendency have particular irritations of the stomach to excite convulsive agitations, that eating certain sweet things has been observed to cause them; swallowing poisonous substances has produced the same effect; and a spasmodic affection of the organs of deglutition

---

* Dr. Coxe is chargable with an oversight in respect to Sydenham's theory of chorea, when he says that Sydenham "scarcely adverts to any thing but its curious gesticulations." Whereas that celebrated physician ascribes the production of chorea to a humour thrown on the nerves, which, by its irritation, occasions such preternatural motions, and accordingly his curative expedients are devised from these two indications: 1, To lessen the humors by bleeding and purging; and 2, To strengthen the nervous system. See *Schedula Monitoria* and *Processus Integra.*

deglutition has been attended with choreatic gesticulations.*

Mr. Alexander, of Montrose, relates three interesting cases of chorea, which were successfully treated by means of zinc, castor, blistering, cold bath, and sedative injections. From the first of those cases it appears, that chorea will sometimes excite paroxysms of muscular exertion as inordinate as are excited by phrenitis, mania, or intoxication, and will thus exhaust the sensorial power so as to produce syncope, followed by strabismus, a symptom of cerebral affection, yet hydrocephalus shall not be the consequence. The strabismus can be with reason ascribed only to the efforts of the brain to recruit its impaired energy; but cannot, on good grounds, be imputed to hydrocephalic oppression. Every circumstance of the case points to diminished tone of the alimentary canal, in a system prone to the operation of morbid irritability, as the occasional cause of the convulsive motions; and consequently it is an impressive example of the tendency which chorea has to produce such changes in the functions of the encephalon, as may ultimately terminate in an aqueous effusion into its cavities. But surely, no theory, even the *newest*, can make us believe, whilst we are in our senses, that every repetition of the strabismus was occasioned by the effusion of a fluid into some cavity of the brain, and that each cessation of the ocular distortion was the effect of the absorption of that fluid.

The second case furnishes an auxiliary to the same side of the question, especially in relation to the cause which generally produces chorea, namely, a morbid condition of the alimentary canal, in a constitution of considerable mobility. The third case supports the second. And they all conspire to shew, that the treatment most likely to succeed, in the cure of chorea, is that which is founded on the tonic and antispasmodic system of prescription. They all likewise tend to evince, that chorea is not a symptomatic ailment, at least not naturally symptomatic of hydrocephalus. On the contrary, it may be termed an idiopathic disease as well as hysteria, whose seat is in the alimentary canal, and which exhibits phenomena more or less

* Edinburgh Medical Commentaries, vol. xii. p. 325. Duncan's Annals of Medicine, 1798, p. 369; Id. 1799, p. 374. London Medical Observations and Inquiries, vol. vi. art. 12.

less convulsive, in proportion to the constitutional excitability of the subject.*

A curious example of convulsive affection, resembling paroxysms of hysteria and chorea combined, is detailed in the New York Repository, Hex. II. vol. i. p. 1—11, and is there ascribed to the bite of a spider, which, like the bite imputed to the tarantula, produced convulsive motions, that were counteracted most effectually by music. This case (the subject a girl fifteen years of age) strengthens the affinity, above suggested, between hysteria and chorea, in regard to the seat of those causes whence they generally originate. Dr. Whytt shews, that a delicate state of the first passages, or a depraved sensibility of their nerves, not only disposes people to many complaints in those parts, but that the whole nervous system is thereby rendered more moveable, and liable to be affected by the slightest exciting causes. Hence tremors, palpitations, convulsive motions, &c. may often be owing more to the infirm state of the first passages, than to any fault in the brain, or in the heart.† And that state of these passages will not be less in the present luxurious and dissipated age, than it was about the middle of the last century.

Dr. Macbride, under the denomination of *Hieranosos*, or *Morbus Sacer*, gives the case of a lad, about seventeen years of age, who at that time had been afflicted with the disorder above twelve years. His body was so distorted, and his legs and arms so twisted round the body, that no words can give an adequate idea of the oddity of his figure; the agitation of the muscles was perpetual; but in general he complained not either of pain or of sickness; and he retained his senses perfectly, insomuch that he used to assist his mother, who kept a little school, in teaching children to read.‡ This appears to be a case of strongly marked idiopathic chorea, without the least morbid affection of the brain.

All the cases of chorea, wherein zinc was tried, in the Bristol Infirmary, from 1784 to 1788, inclusive, were nine, five boys and four girls, seven of whom were cured; at the same time, three cases are subjoined, to show that zinc is not an infallible remedy in this disease. The first case is that of a boy, seventeen years old, who, several years

* Duncan's Annals of Medicine, 1801, p. 303.

† Whytt's Works, p. 537, 550.

‡ Methodical Introduction, p. 558—9.

years before, had received an injury on the head which required the use of the trepan; but who, until three months anterior to the statement of the case, had been very well, and was then attacked with chorea. Zinc, with a decoction of cinchona, during some time, promised a complete recovery; but, after continuing them a month, the disease gradually returned without any known cause, and rose to an extraordinary height. At length, by the administration of opium and camphor, together with a strong preparation of cinchona, the patient was cured. Though, in this case, the convulsive agitations seem to have some distant relation to an externally injured brain, yet it is manifest, from the absence of hydrocephalic symptoms, and from the nature of the remedies, that the state of the brain was totally different from that which exists in hydrocephalus internus, and that those agitations sprung from a morbid action of the nervous influence at large, rather than from a topical congestion in the head. For, if there had been such congestion, strong indications of it would have appeared in so violent a case, and the powerful stimulants, employed in the treatment, would not have had the curative effects.

The other two cases are those of girls, both seven years old. The one was perfectly cured of chorea by zinc, in the space of a few weeks; but, not long afterward, she fell into a hydrocephalus, which proved mortal. This case furnishes a very apposite instance to shew the power which choreatic convulsions have to induce hydrocephalic affection. There is every reason to believe that the original complaint was idiopathic chorea, which was suspended by a tonic mineral remedy; but, having sown the seeds of hydrocephalic action, which, in such subjects, it is very capable of doing, they soon ripened into a destructive maturity. The other girl, growing worse under the use of zinc, was put under a course of cold bathing and cinchona, by which, at the time the paper was written, she was nearly restored to perfect health.*

Five-

---

* Memoirs of the London Medical Society, vol. III. p. 563. Analogous to the first of the above three cases of chorea, a very curious instance is related by Dr. Hall, of East Retford. A woman received a severe blow on the head, which was succeeded by chorea, and yet no symptoms of hydrocephalus appeared throughout a tedious and tormenting spell of the malady. Besides, during the whole course of the disease, stimulants were the principal remedies, and argentum nitratum was reckoned the curative one.

Five instructive cases of chorea, accompanied with judicious remarks, are collected by Mr. M'Mullin, and published in No. I. of the Edinburgh Medical and Surgical Journal. In those cases, it is evident, that the involuntary motions, debility, and other symptoms of the disease, were produced by local irritation in the bowels, which was afterwards communicated to the rest of the system through the medium of the nerves. The disease seldom occurs, except in subjects under the age of puberty, and who are of a delicate constitution. In such habits, irritation may be produced, as Dr. Whytt observed, by very slight causes, such as a depraved state of the intestinal secretions, an accumulation of fæces, worms, dentition, &c. From the appearance of the stools, which were black and fœtid, in Mr. M'Mullin's cases, he thinks it highly probable that the liver was engaged; an idea which derives support from the effects occasioned by the remedies administered. He also thinks, that there was an accumulation of fæces, caused by the use of food too coarse for their weak digestive organs to assimilate, united with a want of that exercise which is necessary for promoting healthy concoctions. Symptoms of the third cause of the disease, specified by Mr. M'Mullin, namely, irritation from worms in the intestinal canal, did not appear in any of the five cases which he transcribes.

Respecting the fourth cause of chorea, cited by Mr. M'Mullin, viz. irritation from teething, he says, that it was first observed by Dr. Monro. "A young lady was affected with chorea of one side. On examining her mouth, it appeared that she was cutting one of the molares of the same side. Dr. Monro directed a crucial incision to be made through the gum, and the disease ceased in a few days. Some months afterwards, she was again seized with chorea, but the other side was affected; and, on examination, it was again found that she was cutting one of the molares of the affected side. A deep crucial incision again removed the disease.* This observation is confirmed by Dr. Gregory. He was called to a boy very ill of chorea, and, on examining his mouth, he found that the second set of teeth was pushing up by the side of the first, which had

---

one. Although, in these two instances, chorea succeeded an external injury inflicted on the head, it will appear, in the sequel, that, in a great majority of such injuries, convulsive affections do not arise

* Dr. Monro, sen. Lectures on Anatomy and Surgery.

had not fallen out at the usual time. By removing a considerable number of the first set, the disease was removed in a very short time, altho', formerly, the spasms had been so violent as to throw him down in the street. Next year he had another attack, which was removed by taking out the rest of the first set of teeth. He was again attacked, however, at the age of fifteen; and as no cause of irritation could be discovered in the mouth, he was treated with extract of cinchona and sulphat of iron, and was cured.

"Another boy, who was twice attacked with chorea, was cured both times by scarifying his gums. Although Dr. Gregory does not attempt to explain why the second set of teeth should thus produce chorea, while the first produces epilepsy, he considers the fact as established, and as forming a very valuable addition to the history, pathology, and treatment of the disease. These important facts directly support the opinion which I have advanced," says Mr. M'Mullin, "that the symptoms of chorea depend on local irritation, and not on debility; and that they are to be removed by removing the causes of irritation, by scarifying the gums, by expelling the worms, or by the use of brisk purgatives." *

The powerful influence of a cause, even indirectly irritating the *primæ viæ*, and thus producing convulsive affections, is strongly exemplified by the history of the poor children, (related by Sir George Baker) who, by breathing vitiated air, were thrown into excessive tormina of the alimentary canal, which were attended with convulsions and delirium. To the same cause, rather than to improper food, may be ascribed the prevalence of chorea in the Orphan Hospital at Milan, as mentioned by Mr. M'Mullin. The principal cause of the *trismus nascentium*, has also been traced to the operation of vitiated air upon the lungs. †

Three extraordinary cases of chronic convulsions, which happened in one family, in the county of Rutland, are related in the Edinburgh Medical Commentaries, vol. ix. p. 317. These cases seem to have been a compound of epilepsy and chorea, as the paroxysms came on periodically and suddenly, beginning with hideous shrieks, suc-

---

* Edinburgh Medical and Surgical Journal, No. I. p. 32.

† London Medical Transactions, vol. III. p. 113. Transact. R. I. A. vol. III. p. 89, 108.

ceeded by jumping, writhing, &c ; but not the smallest symptom of hydrocephalic affection appeared. They first occurred after hooping-cough; and, as the accession of the fits was attended with screaming, it is probable that the painful sensations, whereby they were excited, originated in the lungs, which were affected principally in their father's house, where might have existed some latent effluvia productive of the pulmonic pain.

An herpetic eruption has been known to be attended with racking pain in the stomach, and with general convulsions. In rheumatic fever, epileptic fits have occurred from the stimulus of bile, accumulated in the first passages, and the removal of the bile put a stop to the convulsions.* When the stomach, the centre of sympathy and association, is morbidly affected, no wonder that the nervous system should be engaged, and the moving powers brought into irregular action.

In genuine hydrocephalus internus, convulsive affections do not occur until the disease be far advanced, and the choreatic species of those affections is the rarest at any stage of it. Notwithstanding what Mr. Petit says, I cannot accede to his opinion; for Dr. Whytt, whose authority is of the greatest weight in this question, never saw any convulsions happen till towards the end of the malady. This observation is confirmed by the testimony of numerous practitioners; and, in several cases, not any convulsions have occurred from beginning to end. From a review of thirty two cases of genuine hydrocephalus internus, I find, that, in fifteen of them, no convulsions took place; and that in any of the other seventeen cases, spasms or convulsive motions did not occur, until the hydrocephalus had subsisted several days, nay, in some, not until it had continued several weeks. To these thirty-two cases I can add ten or twelve, running similar courses, which have come within the sphere of my own practice. Besides, from a review of a few cases of spurious hydrocephalus, wherein the head was considerably enlarged, it appears that, in some of these, the convulsions did not attack at the beginning, and that, in others, even in those ascribed to external injuries, no convulsions happened.†

In

---

* Ferriar's Medical Histor. and Reflect. vol. I. p. 18.—Id. vol. II. p. 7, 8.

† Whytt's Works. p. 727.—Edinb. Medic. Comment. vol. VIII. p. 332.—Id.

In a great majority of cases, where wounds have been inflicted on the head by external violence, convulsive affections have not appeared. In forty-three cases, published by Mr. Pott, ten only had convulsions, or spasms; but, in general, not until many days after the accidents. In sixty-two cases, recorded by Mr. O'Halloran, seven only had convulsions, and but two of these had the fits immediately after receiving the injuries; adding these two sums together, the total number will be 105, of which not a sixth part was invaded by convulsions, or spasms. Hence we may fairly conclude, that, where a predisposition in the nervous system is absent, very morbid states of the brain shall exist, without producing convulsive motions.*

What Dr. Coxe styles a *new theory* is nothing more than that pathological doctrine, known since the days of Hippocrates, and termed *Conversion of Diseases.* The conversions of the different genera of fever into each other are common; hæmoptoe is often converted into hysteria, and hysteria into epilepsy, mania, &c.; phthisis pulmonalis is sometimes converted into arthritis, and arthritis into hepatic obstruction, dropsy, lethargy, &c. Hydrocephalus internus has been transmuted into palsy, and palsy into hydrocephalus. Of the latter conversion, Dr. Ferriar furnishes an apposite instance, in which the palsy was evidently connected with the increase of scrophulous swellings on the upper parts of the affected limb. "*Eight months* after the appearance of the paralytic symptoms, the patient complained of severe head-ach, vision became indistinct, and at length was entirely lost. Epileptic fits *then* came on, and he died comatose. When the head

 was

Id. vol. IX. p. 240.—Id. vol. X. 299, 312, 356.—Medical Tracts, vol. I. p. 125, 131.—Edinb. Medic. Comment. vol. XI. p. 298.—Medic. and Physic. Journal, vol. II. p. 131.—Id. vol. III. p. 61, 517.—Id. vol. IV. p. 219.—Id. vol. V. 341.—Id. vol. VIII. p. 98.—Id. vol. X. p. 71, 150—Id. vol. XII. p. 5.—Simmon's Medical Journal. vol. VII. p. 113.—Medic. Observ. and Inquiries, vol. VI.—Art. VI. VII. VIII.—London Medical Transactions, vol. II. p. 358.—Memoirs Medical Society, London. vol. I. p. 165, 169.—Id. vol. II. p. 44.—O'Halloran's Treatise on Disorders from external Injuries of the Head, p. 75.—It is particularly worthy of remark, that this ingenious and able practitioner has anticipated Dr. Gall in the *basis* of his new doctrine of the brain, and the faculties of man. Compare O'Halloran's Treatise, chap. VII. with Dr. Arneman's "Concise Account," Med. and Physic. Journ. vol. XIV. p. 327, 329.

* Potts's Chirurgical Works, vol. I. O'Halloran's Treatise, ch. IV. to XX. p. 36 to 334.

was opened, the ventricles of the brain were found full of water, and several tumours, which, in the prevailing medical language, might be called scrophulous, were observed in different parts of the brain."*

The late Dr. Percival saw a pulmonary consumption converted into a hydrocephalus, by the violent *succussions* of coughing. The patient, a female, nine years old, laboured under the symptoms of phthisis pulmonalis during four months, at the end of which she became affected with unusual pains in the head, that increased rapidly to such a degree, as to excite frequent screamings. "The cough, that had before been extremely violent, and attended with stitches in the breast, now abated; and in a few days ceased almost entirely The pupils of the eyes became dilated; a strabismus ensued; and, in about a week, death put a period to her agonies."†

The case of my patient, mutilated and misconstrued by Dr. Coxe, is a clear case of the conversion of chorea into hydrocephalus, the explanation of which I have already given. It comes under the second subdivision, third head of conversions, thus laid down by Dr. Ferriar. "If the original be a chronic disorder, such a state of the habit (favourable to the production of another disease) may take place during its continuance, and the accessory disease may be simply superadded, or it may vary the form, or affect the duration of the former."‡ Dr. Coxe's first case, which inspired him with the rudiments of the *new theory*, was a case of a similar conversion. The disease subsisted near three months before the Doctor saw the patient, at which time he had little suspicion of water in the brain, as the patient complained of very slight pain in the head at any period after he saw him; neither was the patient affected with strabismus, dilated pupil, nor with any of those changes in the pulse, peculiar to hydrocephalus. And, as the Doctor owns that he "was in a great measure in the *dark*, as to the cause of his illness," I hope he will take in good part my furnishing him with some *light* on the subject. Very much in the dark, indeed, must he have been, and labouring under great perplexity, when we find him expressing his ideas in the following confused and contradictory terms: "The *water* must have been accumulated a con-

---

* Ferriar's Medical Histories and Reflections, vol. II. p. 10, 11.

† Medical Facts, vol. I. p. 131.

‡ Medical Histories and Reflections, vol. II. p. 31.

a *considerable time*, as evinced by the quantity, and the very enlarged state of the foramen ovale. Its *sudden effusion* must have produced apoplexy; but the *slow progress* of the effusion permitted the brain to accommodate itself to the pressure."—" The *water in the brain* was not, I believe, the immediate cause of death. Some *sudden effusion* or congestion was the source of the fatal issue, by producing apoplexy."

In the case of Dr. Coxe's patient, one powerful cause of the conversion of diseases appears to have had full sway, namely, *Medical Treatment*. The unfortunate subject came into the Doctor's hands after undergoing the sufferings of a severe disease, and of vigorous Galenical discipline, nearly three months. In that reduced condition, the Doctor, being most unluckily in the "*dark*," one time bled and purged the patient; another time gave him bark, port-wine, and laudanum; and another, inflamed him with phosphorus. Although every possible effect that this exhausting process could produce was at length obtained; and though the poor victim had lost about an hundred ounces of blood; yet, as the Doctor considered him "labouring under a *depressed* state of the system," an attempt was made to exonerate him by lessening the still oppressive burthen of blood, but, alas! not more than two or three tea-spoonfuls could be extracted from his veins. And, consequently, weighed down with the dire load of an *oppressed system*, that very day the hapless patient sunk into the arms of death?"

" Quot Themison ægros autumno occiderit uno!"

On the practical mischief which would ensue from embracing the *new theory* of Dr. Coxe, I need not particularly enlarge, as it will not be difficult for any of your readers to perceive it by impartially weighing the preceding facts and observations. But, in respect to medical science, there is a mischief to which that theory ministers, viz. the propensity to generalize without sufficient data, which requires some distinct animadversion. This propensity, under the pretence of *simplifying*, may be accused of doing harm not only to medical science in particular, but also to science in general. The immortal Verulam, to whom genuine philosophy is highly indebted, maintains this opinion: " Solent autem homines naturam," says that truly great man, " tanquam ex præalta turri, et a longe despicere, et circa *generalia* nimium occupari, quando si descendere placuirit, et ad *particularia* accedere, resque ipsas at-

 tentius tentius et diligentius inspicere, magis *vera* et utilis fierit comprehensia. Ideoque," continues he, dubitandum non est quin si *Medici,* missis paulisper istis *generalibus,* naturæ obviam ire vellent; compotes ejus fierent, de quo ait poeta,

> "Et quoniam variant morbi, variabimus artes;

> Mille mali species, mille salutis erunt."

This doctrine is enforced, in a very sensible manner, by Mr. M'Mullin, at the beginning of the paper already quoted, where he says, "Few circumstances have contributed more to retard the progress of the healing art than premature generalization. Our opinions are often derived from the consideration of insulated facts, which have accidentally made a strong impression on our minds. The partial views thus obtained, from our impatience to arrive at general principles, we immediately assume as the basis of a system which we believe to be incontrovertible; because with unconscious partiality, we exaggerate the importance of every argument which seems to support it, and neglect or distrust every fact with which it is incompatible."

Thus, when men infer general theorems and universal propositions from a few experiments or observations, it is logically termed a *false induction*; to which sort of sophism, Dr. Coxe, on this occasion, appears to be too much addicted; and in drawing a parallel between chorea and hydrocephalus, he is no less prone to resort to fallacious argument. There we find him trying every sophism, which his art of logic can furnish, such as the *petitio principii*, the *non causa pro causa,* and the *fallacia accidentis.* Nor is he more observant of the canons of pathology, than he is of the laws of reasoning. According to his pathology, chorea is merely a symptom of hydrocephalus, and yet, in his parallel, he reckons that *single symptom* similar to the *whole disease.*

The self-complacency, which Dr. Coxe discovers in conferring on his arbitrary proposition the title of "A New Theory," brings to my recollection an instance of a similar nature with respect to the structure of language. A gentleman of some talents, but little acquainted with the progress of letters, composed an English grammar which he conceived to be *perfectly new,* and as such, he shewed it to a friend, who was more conversant with books than he was, but who, unluckily for his hopes, convinced him that he had been anticipated by Louth. Such a friend and confidant would have been an acquisition to Dr. Coxe. If no such person were attainable, he might have found a useful monitor in a very a very old writer, of unquestionable authority, (provided he ever peeps into old works), who has pronounced, "The thing that hath been, it is that which shall be; and that which is done, is that which shall be done: and there is *no new thing* under the sun." This being the decision of a wise and good man, whose authority we must reverence and should not oppose, it is applied, as an *argumentum ad verecundiam*, or an address to the *modesty* of Dr. Coxe, by

Yours, &c.

*Londonderry, Dec.* 16, 1805. Wm. PATTERSON.

